# *Fusarium* and *Neocosmospora* Species Associated with Rot of *Cactaceae* and Other Succulent Plants

**DOI:** 10.3390/jof8040364

**Published:** 2022-04-01

**Authors:** Sahar Kamali-Sarvestani, Reza Mostowfizadeh-Ghalamfarsa, Fatemeh Salmaninezhad, Santa Olga Cacciola

**Affiliations:** 1 Department of Plant Protection, School of Agriculture, Shiraz University, Shiraz 7144165186, Iran; SaharKamali1373@gmail.com (S.K.-S.); f.Salmaninezhad@shirazu.ac.ir (F.S.); 2 Department of Agriculture, Food and Environment (Di3A), University of Catania, 95123 Catania, Italy

**Keywords:** *Cactaceae*, *Nectriaceae*, *Fusarium oxysporum* f. sp. *opuntiarum*, *Fusarium proliferatum*, *Neocosmospora falciformis*, phylogenetic analysis, *tef1* gene, pathogenicity, cross-inoculations, host range

## Abstract

Infections by *Fusarium* and *Fusarium*-like species on cacti and other succulent plants cause the syndrome known as Fusarium dry rot and soft rot. There are only few records of *Fusarium* species as pathogens of cacti and other succulent plants from Iran. The objective of this study was the identification and characterization of fusarioid species recovered from ornamental succulents in Shiraz County, Iran. Three fusarioid species, including *F. oxysporum*, *F. proliferatum*, and *Neocosmospora falciformis* (formerly *F. falciforme*), were recovered from 29 diverse species of cacti and other succulents with symptoms of Fusarium dry rot and soft rot. The three fungal species were identified on the basis of morphological characters and the phylogenetic analysis of the translation elongation factor1-α (*tef1*) nuclear gene. The *F. oxysporum* isolates were identified as *F. oxysporum* f. sp. *opuntiarum*. The pathogenicity of the three fusarioid species was tested on a range of economically important ornamental succulents, mostly in the *Cactaceae* family. The three species showed a broad host spectrum and induced different types of symptoms on inoculated plants, including soft and dry rot, chlorosis, necrotic spots, wilt, drying, root and crown rot. This is the first report of *N. falciformis* as a pathogen of succulent plants worldwide.

## 1. Introduction

There are diverse definitions of succulent plants [1]. In accordance with an inclusive concept, succulent plants are characterized by fleshy tissues adapted to water storage. Some succulents, such as cacti, store water in the stem, whereas others store water mainly in the leaves. Most succulents are native to arid regions and consequently show some degree of xerophytic adaptation, such as a modified cycle of CO_2_ fixation called crassulacean acid metabolism. Succulent plants are found in more than 60 plant families, including *Aizoaceae*, *Asclepiadaceae*, *Crassulaceae*, *Euphorbiaceae*, and *Cactaceae*, to cite the most numerous ones [2,3,4,5]. The *Cactaceae* family comprises four subfamilies, 127 genera, and more than 2000 species [6]. Species in this family are economically relevant as ornamental, landscape, medicinal, and crop plants [7,8]. Succulent plants are susceptible to various plant pathogens [9,10,11,12]. Among them, soil-borne fungi of the genus *Fusarium* and its allied genera stand out for the severe losses they cause on ornamental cacti in greenhouse [11].

The identification of *Fusarium* species is challenging, as the taxonomy and nomenclature of this genus has been in a continuous flux since its first description. Recently, phylogenetic criteria were introduced to revise the taxonomy of this genus, and as a consequence, a more restricted concept of *Fusarium* was defined, and new genera, such as *Neocosmospora*, were segregated from *Fusarium sensu lato* [12,13]. However, there are conflicting opinions and an ongoing, lively debate on whether to keep these new genera separate from *Fusarium sensu stricto* or merge all fusarioid species in a single genus, conforming with a broad concept of *Fusarium* [14,15,16]. Translation elongation factor1-α (*tef1*) was shown to be a suitable barcode for resolving species of *Fusarium* and allied genera [13]. The definition and identification of *formae speciales* (ff. spp.) of *Fusarium* are even more challenging [17]. The concept of *forma specialis* (f. sp.) was initially restricted to a single plant species and subsequently was broadened to include strains with the same host range [17]. *Fusarium oxysporum* f. sp. *opuntiarum* (Pettinari) W.L. Gordon, for example, is one of the more than 140 recognized *formae speciales* of *F. oxysporum* von Schlechtendal emend. Snyder and Hansen; presently, its host range encompasses succulent plants in the family *Cactaceae* and *Euphorbiaceae*, including *Astrophytum myriostigma* Lem., *Cereus* spp., *Echinocactus grusonii* Moran, *Espostoa lanata* (Kunth) Britton and Rose, *Euphorbia mammilaris* L., *Ferocactus latispinus* (Haworth) Britton and Rose, *Mammillaria zeilmanniana* Boed., *Opuntia ficus-indica* (L.) Mill. and *Schlumbergera truncata* Moran [11,17]. Beside *F. oxysporum* f. sp. *opuntiarum*, other *Fusarium* ff. spp. and species were reported as pathogens of succulent plants, including *F. oxysporum* f. sp. *crassulae; F. oxysporum* f. sp. *echeveriae*; *F. equiseti* (Corda) Sacc.; *F. fujikuroi*, the anamorph of *Giberella fujikuroi* (Sawada) Wollenw.; *F. lunatum* (Ellis and Everh.) Arx, now *Bisifusarium lunatum* (Ellis and Everh.) L. Lombard and Crous; and *F. proliferatum* (Matsush.) Nirenberg ex Gerlach and Nirenberg, a species in the *F. fujikuroi* species complex (FFSC) [9,18,19,20,21]. There are also reports of *F. oxysporum* as a pathogen of succulents, such as *Agave tequilana* F.A.C. Weber (family *Asparagaceae sensu lato*), *Aloe barbadensis* Miller (family *Aloeaceae*), *Echinopsis oxygona* Link. (Zucc.) ex Pfeiff. and Otto (family *Cactaceae*), and *Hylocereus undatus* (family *Cactaceae*), but without any evidence of a f. sp. involved [22,23,24,25].

*Neocosmospora* (*Hypocreales*, *Nectriaceae*), quite recently segregated as a distinct genus from the *F. solani* species complex (FSSC), includes ubiquitous fungi with a worldwide distribution that are usually found in soil, plant debris, living plant material, air, and water. It comprises both plant and human pathogens and has been isolated from nearly 500 different host plants of more than 100 families [12,13,14,15,16].

Fusarioid species survive in the soil as chlamydospores or mycelium associated with plant debris and organic matter. Although behaving prevalently as soil-borne pathogens, they may infect above ground organs of the plant by aerially dispersed conidia. Symptoms incited by fusarioid species on succulent plants are of diverse types, including soft and dry rot, root and crown rot, stem rot, chlorosis, and necrotic spots, and they are collectively referred to as Fusarium rot [11,26].

From 2017 to 2018, *F. oxysporum* isolates were recovered from infected tissues of succulent plants, such as *Cereus* sp., *Echinocactus* sp., *Ferocactus* sp., *Notocactus* sp. and *Opuntia* sp., under greenhouses in Iran. Subsequently, these isolates were identified as *F. oxysporum* f. sp. *opuntiarum* [27]. In the last years, symptoms suggestive of Fusarium soft and dry rot have frequently been observed on several cacti and other succulent plants in commercial greenhouses for the production of ornamentals in Shiraz County, Fars Province, Iran. The main objectives of this study were to identify the *Fusarium* and *Fusarium*-like species associated with these symptoms and to investigate the ability of these fungi to infect different species of succulent plants.

## 2. Materials and Methods

### 2.1. Isolation of Fungi

To identify the causal agents of Fusarium rot of succulent plants in commercial greenhouses for the production of ornamentals in Shiraz County, during 2017–2018, infected root crown and stem tissues from symptomatic potted plants were collected in the municipalities of Shiraz, Bajgah, and Sadra. The coordinates of sampling sites were recorded by Global Positioning System (GPS) (Table 1). Root and rhizosphere soil samples were brought to the Mycology Laboratory of the Department of Plant Protection, Shiraz University. To isolate fusarioid species, root and symptomatic crown tissues were put into Erlenmeyer flasks containing sterile distilled water (SDW) on a shaker device for 30 min, and the water was replaced every 10 min. After washing, the tissues were immersed in sodium hypochlorite 0.5% for 10 s, and after re-washing with SDW for 5 to 6 h, they were placed on sterile paper towels for drying. Then, they were cut into small segments or blocks (5 mm) and placed in Petri dishes on Potato-Dextrose-Agar medium amended with streptomycin (PDA; Oxoid Ltd., Basingstoke, UK; streptomycin 200 µg/mL) [28]. After 3 to 5 days incubation at 24 °C, mycelium plugs were transferred to water agar medium (WA; agar 10 g/L). Isolates were purified by single-spore subculture on WA according with a standard protocol [29] and stored on PDA slopes at 4 °C.

### 2.2. Morphological Characterization

All isolates were characterized based on their cultural and morphological characteristics. Colony morphology, pigmentation, and type of aerial mycelium were determined on PDA. Morphological observations included the presence and characteristics of sporodochia, size of sporodochial (macro-) and aerial (micro-) conidia, shape and degree of septation of conidia; disposition of the microconidia; and conidiophore length and branching patterns, type of the conidiogenous cells, and presence or absence of chlamydospores, according with standard protocols [30,31]. To produce sporodochia, agar blocks from single-spore cultures were placed in Petri dishes (60 mm diameter) on carnation leaf-piece agar (CLA) medium prepared according to the Fusarium Laboratory Manual [32]. Carnation leaves were cut into pieces (5 × 5 mm), dried at 60 °C for 24 h, sterilized by autoclaving, and placed on nearly solid 1.5% WA. Petri dishes were incubated at 25 °C for 7–14 d under 12 h cool fluorescent light/dark cycles. To produce microconidia, cultures were prepared on KCLA (carnation leaf agar supplemented with 8 g/L of potassium chloride) [33] agar medium by transferring agar blocks of 5 × 5 mm from cultures grown on CLA [32] immediately after the production of sporodochia. Chlamydospore formation was checked on cultures growing on PDA, CLA, KCLA, and synthetic nutrient-poor agar (SNA) [32,33] with and without sterilized pieces of carnation leaves, incubated at room temperature with a 12 h cool fluorescent light/dark photoperiod [32,34,35]. Slide preparations for microscopical observations were mounted in water. Isolates were identified using morphological identification keys [32,36].

### 2.3. DNA Extraction, PCR, Sequencing, and Phylogenetic Analyses

A more restricted number of selected isolates was identified by phylogenetic analysis of the translation elongation factor1-α (*tef1*) nuclear gene. The DNA was extracted from 0.5 × 0.5 mm fungal blocks from 5-day-old colonies on PDA according to the protocol of Schena et al. [37]. The extracted DNA was kept at −20 °C for further studies. DNA quality was examined with an MD-1000 Nanodrop spectrophotometer (Nanodrop Technologies, DE, USA) [37]. The translation elongation factor1-α (*tef1*) gene was amplified with the pair primers TEF1 (5′ATGGGTAAGGARGACAAGAC3′) and TEF2 (5′GGARGTACCAGTSATCTG3′) according to O’Donnell et al. [38]. The amplification conditions for *tef1* were: 95 °C for 3 min, 35 cycles for 94 °C for 60 s, 55 °C for 30 s, 72 °C for 90 s, and 72 °C for 10 min. The PCR products were sequenced with the primers used for amplification by a dye terminator cycle (Shahid Rajaie Cardiovascular, Medical, and Research Center, Tehran, Iran). The resulting sequences were submitted to GenBank and acquired accession numbers are listed in Table 2. Raw sequences were edited by BioEdit [39], and sequence alignment was performed by Clustal X with subsequent visual adjustments [40].

*Geejayessia atrofusca* (Schwein.) Schroers and Gräfenhan (accession No. AF178361) was considered as an outgroup for *Fusarium* spp. and *Neocosmospora* spp. phylogenetic trees. To reconstruct the phylogenetic trees, Bayesian inference analyses on *tef1* locus were carried out with MrBayes v. 3.1 [41], imposing a general time-reversible (GTR) substitution model with gamma (G) and proportion of invariable site (I) parameters to accommodate variable rates across sites. Bayesian analyses were conducted with the same data set according to Salmaninezhad and Mostowfizadeh-Ghalamfarsa [42]. The best nucleotide substitution model was determined by MrModelTest v. 2.3 [43] Two independent runs of Markov chain Monte Carlo (MCMC) using four chains were run over 1,000,000 generations. Trees were saved each 1000 generations, resulting in 10,001 trees. Burn-in was set at 5% generations. The phylogenetic tree was constructed using TrEase [44], and the resultant tree was edited and displayed with Mega 7.1 [45]. Alignments and trees were submitted to TreeBASE [46].

### 2.4. Pathogenicity Tests and Host Range

The same 13 isolates identified at the species level by phylogenetic analysis of the *tef1* nuclear gene were evaluated for their ability to infect succulent plants. To perform pathogenicity tests, two distinct methods of inoculation were compared. In the first method, 1 mL of a conidial suspension (10^6^ conidia per mL) was injected into each plant. Control plants were injected with SDW [27,47].

In the second method, 50 mL of autoclaved wheat seeds were placed into 250 mL Erlenmeyer flasks and inoculated with mycelium plugs (three 5 mm mycelium plugs per flask). After two weeks incubation at 25 °C, the resulting inoculum was used for root inoculation of test plants by removing the soil around the crown and placing 10 seeds colonized by the pathogen at the base of the stem. The seeds were then covered with the soil [48]. Control plants were inoculated with sterile seeds.

Potted 2- to 3-year-old plants were used in pathogenicity tests, including species of *Aeonium*, *Astrophytum*, *Braunsia*, *Carnegiea*, *Cephalocereus*, *Cereus*, *Echeveria*, *Echinocactus*, *Echinocereus*, *Ferocactus*, *Gymnocalycium*, *Hamatocactus*, *Mammillaria*, *Notocactus*, *Opuntia*, *Sedum*, and *Sempervivum*. Plants were grown in greenhouse at temperature ranging from 22 to 26 °C and were examined for the presence of symptoms up to 40 days post inoculation (d.p.i.).

## 3. Results

### 3.1. Sampling

Overall, 29 species of succulent plants belonging to 10 diverse genera of the families *Cactaceae* and *Crassulaceae* and showing symptoms of crown and root rot were sampled during the survey (Table 1). Basically, the symptoms were of two types: dry rot and soft rot. Dry rot was prevalent on species of *Aeonium*, *Echeveria*, *Ferocactus*, *Mammillaria,* and *Notocactus*, while soft rot was mainly observed on species of *Astrophytum*, *Cephalocereus*, *Echinocactus*, *Echinocereus*, and *Gymnocalycium*. A total of 62 fusarioid isolates were obtained from symptomatic plants. Their hosts and geographic origin are reported in Table 1.

### 3.2. Morphological Characterization of Isolates of Fusarium and Neocosmospora Species

**Figure 1:**
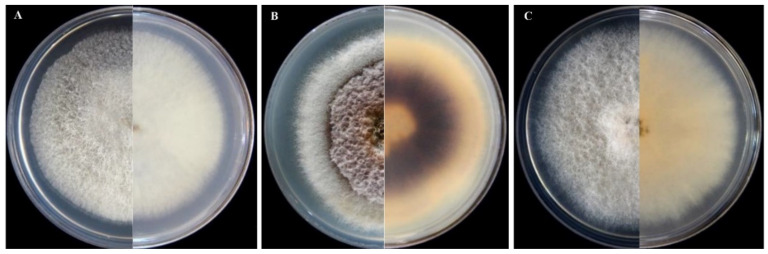
Colony morphology of (**A**) *Neocosmospora falciformis* (isolate FNol01); (**B**) *Fusarium oxysporum* f. sp. *opuntiarum* (isolate OEcg42); and (**C**) *Fusarium proliferatum* (isolate PEcg29) from succulent plants; front (left) and back (right) side after 5 days incubation on PDA at 25 °C in the dark.

Despite the variability in colony morphology and micromorphological traits as well as the partial overlapping of the dimensions of micro- and macroconidia, the 62 fusarioid isolates obtained from succulent plants could be grouped into three distinct morphotypes. The most numerous group encompassed 38 isolates recovered from plants of the family *Cactaceae* and *Crassulaceae*. These isolates formed on PDA colonies that were fast-growing, uniform, with aerial mycelium denser in the center of the colony, and a diffuse pale pink pigmentation, which turned dark violet with age (Figure 1B). Sporodochial conidia produced on CLA were three septate, with mean dimensions of 30.95 × 2.84 μm (range 27 to 36 μm in length and 3.0 to 4.0 μm in width). Measures were taken on 30 macroconidia per isolate. Microconidia were oval to obovoid in shape, non-septate; conidiophores were monophialidic and chlamydospores single or double. A second morphotype comprised 17 isolates recovered from plants of the families *Cactaceae* and *Crassulaceae*. They formed on PDA colonies that were fast-growing, uniform with aerial mycelium, and a diffuse yellowish pigmentation fading to pale pink (Figure 1C). Sporodochial conidia produced on CLA were three to four septate with mean dimensions of 45.6 × 6.0 μm (range 5.1 to 41.7 μm in length and 3.2 to 9.5 μm in width). Measures were taken on 30 macroconidia per isolate. Microconidia were ellipsoid to oval in shape, 0 to 1 septate; conidiophores were prevalently monophialidic and less frequently polyphialidic; chlamydospores were absent in some isolates, while in others, they were produced in a large amount and were single-celled, terminal, and typically rough-walled. A third and less numerous group comprised seven isolates, all recovered from plants of the family *Cactaceae*. Colonies of these isolates growing on PDA were fast growing, floccose, and white, with no evident pigmentation (Figure 1A). Sporodochial conidia produced on CLA were straight to falcate, moderately curved and slender, sometimes strongly curved, with apical cell papillate and basal cell foot-shaped to barely notched, one to four septate, with mean dimensions of 45.6 × 3 μm (range 16.5 to 55 μm in length and 2 to 4.5 μm in width), and clustering in discrete false heads at the tip of phialides. Measures were taken on 30 macroconidia per isolate. However, a few isolates of this morphotype failed to produce sporodochia. Microconidia were ovoid to pear-shaped, prevalently non septate, and rarely one septate; sporodochial conidiogenous cells were mono- and polyphialidic; chlamydospores were absent.

Based on these morphological characteristics, the second and third groups of isolates were tentatively identified as *Neocosmospora falciformis* (Carrión) L. Lombard and Crous, formerly *F. falciforme* (Carrión) Summerb. and Schroers, and *F. proliferatum* (Matsush.) Nirenberg, respectively, while the first and most numerous groups was tentatively identified as *F. oxysporum* f. sp. *opuntiarum* W.L. Gordon, based on both morphological traits and the range of naturally infected host plants from which isolates were recovered, prevalently encompassing the species of *Cactaceae*.

### 3.3. Phylogenetic Analysis

**Figure 2:**
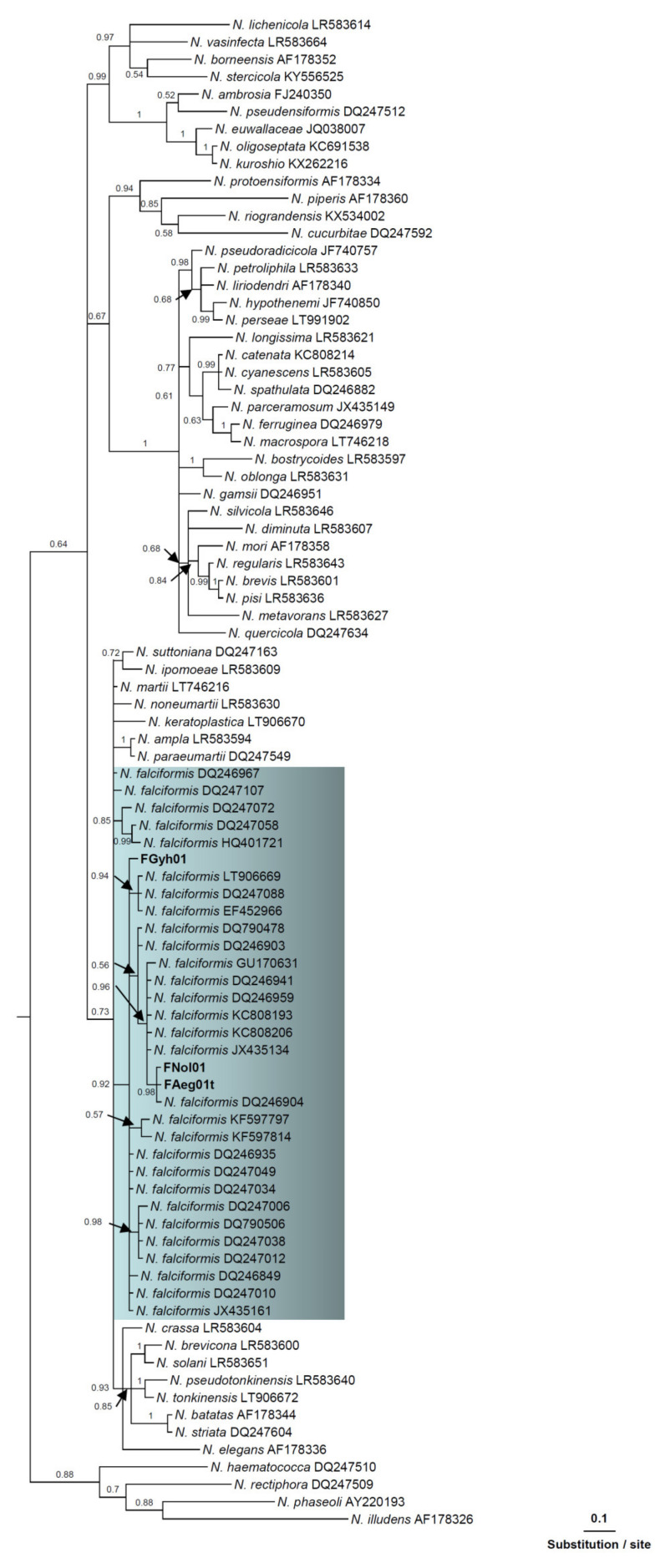
Phylogenetic relationships of *Neocosmospora falciformis* isolates recovered from Shiraz County greenhouses with other *N. falcifomis* isolates and 56 diverse *Neocosmospora* species (see Sandoval-Denis et al. 2019) based on Bayesian analysis of translation elongation factor1-α (*tef1*) sequences. Numbers above the branches represent the posterior probability based on Bayesian analysis. Isolates retrieved from succulent plants in Iran are shown in bold.

**Figure 3:**
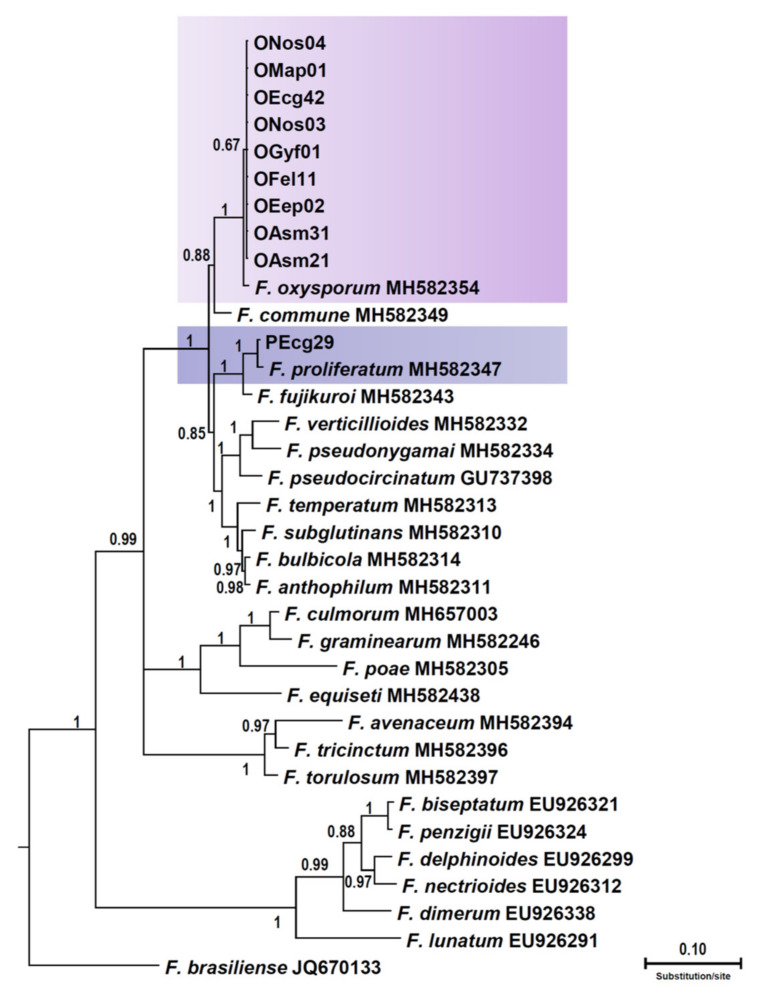
Phylogenetic relationships of *Fusarium* species recovered from Shiraz County greenhouses with 24 *Fusarium* species based on Bayesian analysis of translation elongation factor1-α (*tef1*) sequences. Numbers above the branches represent the posterior probability based on Bayesian analysis. Isolates retrieved from succulent plants in Iran are shown in bold (or arrows point at isolates retrieved form succulent plants in Iran).

Thirteen isolates, nine of the morphotype identified tentatively as *F. oxysporum* f. sp. *opuntiarum*, one from the morphotype identified tentatively as *F. proliferatum*, and three from the morphotype identified tentatively as *N. falciformis*, were selected for the phylogenetic analysis based on the translation elongation factor1-α (*tef1*) gene sequences. Molecular diagnosis confirmed unequivocally the identification based on morphological traits. The final alignment length was 647 bp. Each new group of isolates formed a monophyletic group in Bayesian trees (Figure 1 and Figure 2). The Bayesian posterior probability for each lineage ranged from 0.52 to 1.00. The *tef1* sequences of the isolates FNol01, FGyh01, and FAeg01 clustered with reference *N. falciformis* isolates [13].; the sequences of isolates OMap01, OGyf01, OEep02, ONos03, ONos04, OFel11, OEcg42, OAsm31, and OAsm21 clustered with *F. oxysporum* f. sp. *opuntiarum* (MH582354, [16]); and PEcg29 clustered with *F. proliferatum* (MH582347, [16]), with a high posterior probability of 0.52 to 1.00 (Figure 2 and Figure 3).

### 3.4. Pathogenicity Tests and Host Range

On homologous test plants, all thirteen selected fungal isolates of the three identified fusarioid species (nine of *F. oxysporum* f. sp. *opuntiarum*, one of *F. proliferatum*, and three of *N. falciformis*) incited the same symptoms as those observed on plants with natural infections sampled in commercial greenhouses. Moreover, the isolates induced symptoms on numerous other artificially inoculated succulent plants (Table 2). Conversely, all control plants remained symptomless.

**Figure 4:**
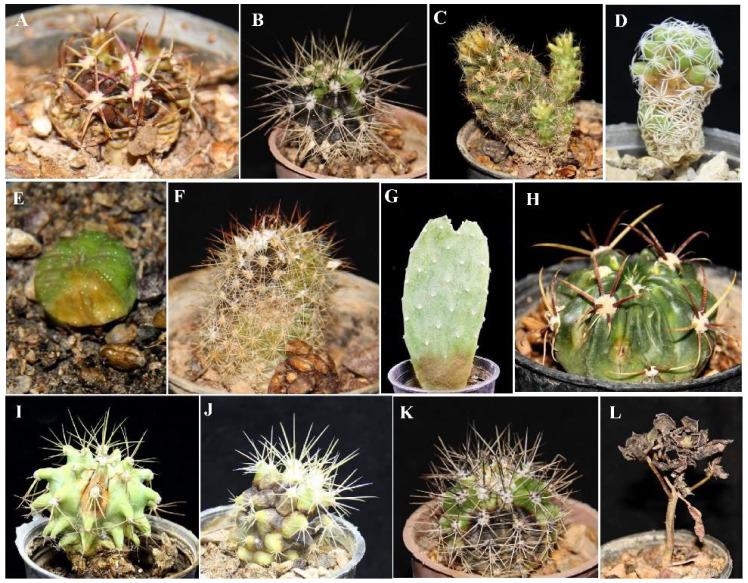
Symptoms induced by artificial inoculation of *Neocosmospora falciformis* on various succulent plants. (**A**) Crown rot on *Ferocactus macrodiscus*; (**B**) root and crown rot on *Mammillaria* *bernalensis*; (**C**) brown rot on *Mammillaria prolifera*; (**D**) root and crown rot on *Mammillaria gracilis*; (**E**) root and stem rot on *Astrophytum asterias*; (**F**) rotting, yellowing, and black spots on *Mammillaria spinosissima*; (**G**) crown rot on *Opuntia ficus-indica*; (**H**) chlorosis on *Ferocactus macrodiscus*; (**I**) chlorosis on *Ferocactus glaucescens*; (**J**) black spots on *Echinocactus grusonii*; (**K**) necrosis and black spots on the crown of *Mammillaria bernalensis*; and (**L**) root and crown rot on *Aeonium arboreum*.

**Figure 5:**
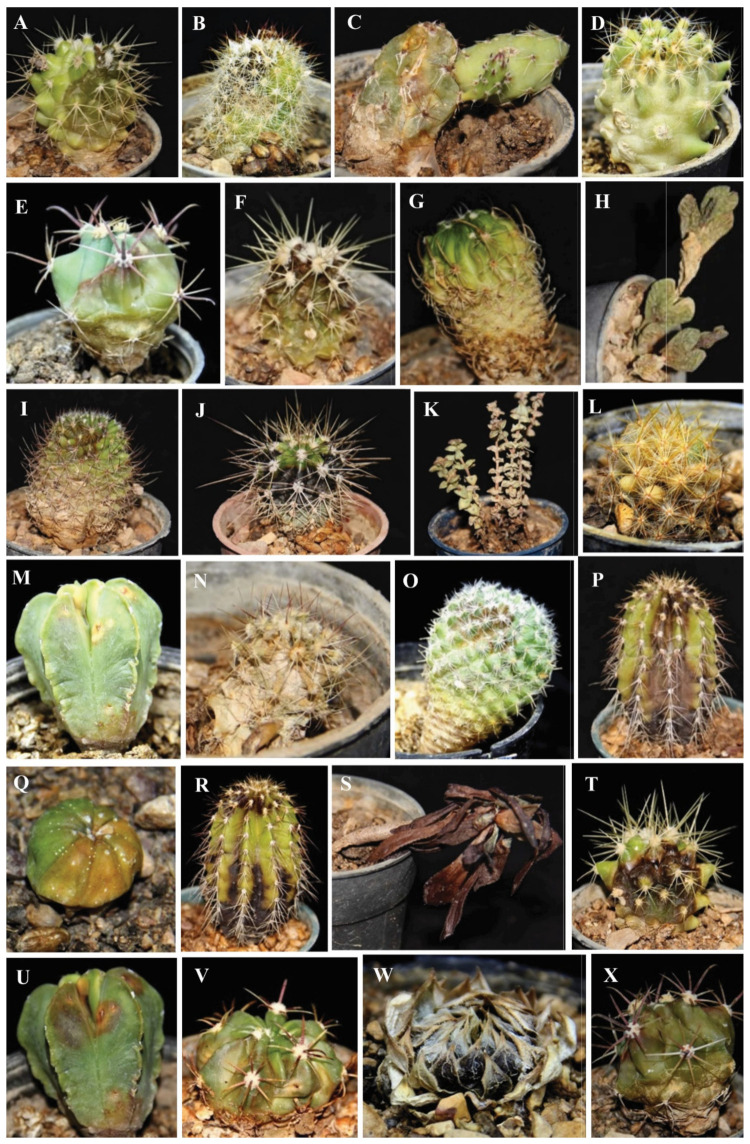
Symptoms induced by artificial inoculation of *Fusarium oxysporum* f. sp. *opuntiarum* on different succulent plants. (**A**–**C**) necrosis and yellowing on *Echinocactus grusonii*, *Mammillaria spinosissima,* and *Opuntia fragilis*, respectively; (**D**) discoloration and rotting on *Hamatocactus setispinus* (syn. *Thelocactus setispinus*); (**E**) root and crown rot with yellowing on *Ferocactus emoryi*; (**F**) rot and death of *Echinocactus grusonii*; (**G**) yellowing, discoloration, and crown rot on *Stenocactus multicostatus*; (**H**) root and crown rot as well as leaf dessication on *Braunsia apiculata*; (**I**) crown rot and yellowing on *Mammillaria jaliscana*; (**J**) root and crown rot on *Mammillaria bernalensis*; (**K**) root and crown rot on *Sedum reflexum* “Angelina”; (**L**) root and crown rot, yellowing, and necrotic area on *Mammillaria gracilis*; (**M**) yellowing and chlorosis on *Astrophytum myriostigma*; (**N**) root rot on *Echinocactus grusonii*; (**O**) crown rot, yellowing, and chlorosis on *Mammillaria matudae*; (**P**) dark-brown spots and soft rot on *Carnegiea polylopha* (syn. *Neobuxbamia polylopha*); (**Q**) soft rot on *Astrophytum asterias*; (**R**) black spots and stripes on *Carnegiea polylopha*; (**S**) root and crown rot on *Aeonium arboreum*; (**T**) black spots on crown and stem of *Echinocactus grusonii*; (**U**) root and crown rot, yellowing, and chlorosis on *Astrophytum myriostigma*; (**V**) chlorosis on *Ferocactus macrodiscus*; (**W**) root rot and drying on *Sempervivum tectorum*; and (**X**) root and crown rot as well as yellowing on *Ferocactus emoryi*.

**Figure 6:**
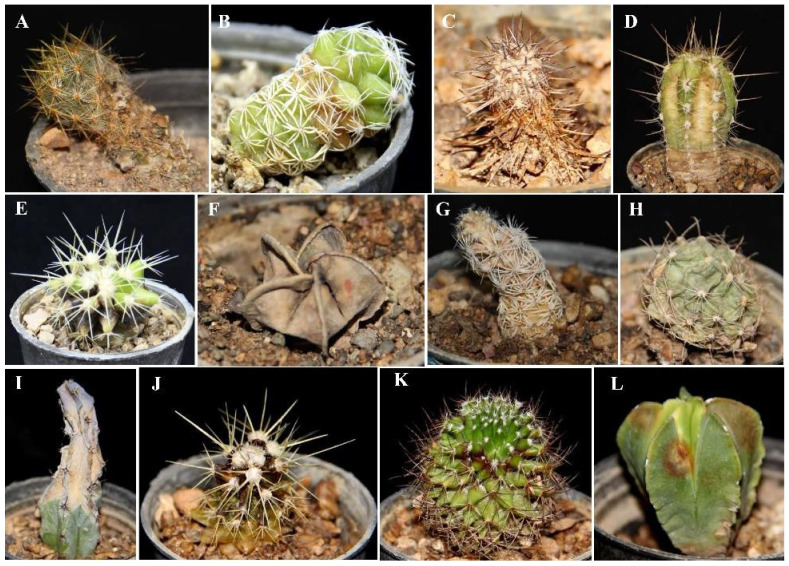
Symptoms induced by artificial inoculation of *Fusarium proliferatum* on different succulent plants. (**A**) rotting, yellowing, and girdling of the basal stem in *Mammillaria prolifera*; (**B**) root and crown rot on *Mammillaria gracilis*; (**C**) plant decline and death on *Mammillaria pottsii*; (**D**) crown rot and yellowing on *Cephalocereus euphorbioides*; (**E**) black spots on crown and stem of *Echinocactus grusonii*; (**F**) crown rot and yellowing on *Astrophytum myriostigma*; (**G**) crown rot and yellowing on *Mammillaria gracilis*; (**H**) basal sunken lesion and root rot in *Hamatocactus setispinus* (syn. *Thelocactus setispinus*); (**I**) plant decline from the top of the stem on *Cereus jamacaru*; (**J**) crown rot and yellowing on *Echinocactus grusonii*; (**K**)crown rot and yellowing on *Mammillaria jaliscana*; and (**L**) chlorosis and soft brown spots on *Astrophytum myriostigma*.

The inoculated fungi were reisolated from symptomatic plants, thus fulfilling Koch’s postulates. Different types of symptoms were observed, such as black to dark-brown spots, yellowing and chlorosis, stunting, apical necrosis, brown to black necrotic areas on the stem, internal rotting, loss of turgor, and stem crinkle leading to plant death (Figure 4, Figure 5 and Figure 6). On all injected plants, first symptoms of rot appeared 10 days post inoculation (d.p.i.) with the only exception of *Mammillaria gracilis* and *M. prolifera* plants, inoculated with *F. proliferatum,* which showed first symptoms 7 d.p.i. Injected plants of these two *Mammillaria* species died within 10 d.p.i., while injected plants of other succulent species, including *M. bernalensis*, *M. jaliscana*, *M. pottsii*, and *M. spinosissima*, died between 16 and 30 d.p.i. All plants inoculated with the three fusarioid species through the soil showed first rot symptoms 20 d.p.i., once again with the exception of *M. gracilis* and *M. prolifera* plants, inoculated singularly with *F. oxysporum* f. sp. *opuntiarum* and *F. proliferatum*, which showed the first symptoms 15 d.p.i., confirming differences in susceptibility to Fusarium rot among tested succulents.

## 4. Discussion

Three species, namely *F. oxysporum* f. sp. *opuntiarum*, *F. proliferatum*, and *N. falciformis*, were found to be responsible for symptoms of dry and soft rot on succulent plants sampled during the survey of greenhouses in Shiraz County aimed at characterizing the diversity of fusarioid species associated with this disease. All three fungal species showed a wide range of natural hosts and an even broader spectrum of potential hosts, as shown by the results of artificial inoculations. The most common and polyphagous species was *F. oxysporum* f. sp. *opuntiarum*, a well-known pathogen of *Cactaceae* with a worldwide distribution, previously reported also in Iran but on a narrower range of host plants [17,27,49]. This f.sp. has been also reported as a pathogen of *Euphorbia mammillaris* var. *variegata*, a succulent plant of the family *Euphorbiaceae* [11]. Results of the present study further broaden the range of potential host plants of *F. oxysporum* f. sp. *opuntiarum*, extending it to members of the family *Crassulaceae* and *Aizoaceae*. Moreover, two other ff. spp. of *F. oxysporum*, f. sp. *crassulae*, and f. sp. *echeveriae*, both with a more restricted host range than *F. oxysporum* f. sp. *opuntiarum*, were reported as pathogens of *Crassulaceae* species [19,20]. This provides further evidence that the definition of f. sp. *opuntiarum* as well as of other *F. oxysporum* ff. spp. with a broad host range, based originally on host specificity and host range, is questionable and of limited diagnostic value if it is not supported by molecular and phylogenetic criteria [11,17,50,51,52]. The definition of ff. spp. of *F. oxysporum* in particular has become more challenging after the demonstration that some of them are polyphyletic [52,53,54,55].

*Fusarium proliferatum*, a polyphagous species whose host range spans from plants to animals [56,57], was already known as a pathogen of *Cactaceae*, having been reported as causal agent of stem rot and soft rot of *Hylocereus polyrhizus* (Weber) Britton and Rose and *Echinopsis chamaecereus* H. Friedrich and Glaetzie, respectively [18,58]. In this study, *F. proliferatum* was recovered from symptomatic plants of *A. myriostigma*, *E. grusonii*, *F. gatesii*, *M. prolifera*, *M. vetula*, and *N. rutilans*. Moreover, including plants that were susceptible to artificial infections, the host range of this *Fusarium* species also comprised *C. euphorbioides*, *C. jumacaru*, *H. setispinus*, *M. gracilis*, *M. jaliscana*, and *M. potsii*, all belonging to the family *Cactaceae*. None of these plants was previously reported as a host of *F. proliferatum*.

In the phylogenetic tree based on *tef1* sequences, *N. falciformis* isolates recovered from succulent plants in Shiraz County clustered with diverse *N. falciformis* isolates from other studies [13,14,16,59]. According with the classification of the 80 species of the FSSC proposed by Geiser et al. [60], *F. falciforme*, the former name of *N. falciformis*, is a member of Clade 3, the largest clade of this species complex. *Neocosmospora falciformis*, besides being a plant pathogen, is a clinically relevant fungus capable of inducing disease on humans and animals generally as an opportunistic pathogen [13]. However, it includes also aggressive plant pathogens, such as the former *F. paranaense*, a species responsible for root rot of *Glycine max* in Brazil, which has been reduced in synonymy with *N. falciformis* after the phylogenetic revision of the genus *Fusarium* [13,61]. Moreover, under the name of *F. falciforme*, *N. falciformis* was reported as a pathogen of *Phaseolus lunatus* and was identified as one of the species associated with Fusarium wilt of *Cannabis sativa* and wilt and bud rot of *A. tequilana* [57,62,63]. The host range of *N. falciformis* isolates recovered in this study from naturally infected plants encompassed plant species of both *Cactaceae* and *Crassulaceae* families, including *Ae. gomerense*, *C. euphorbioides*, *E. gibbiflora*, *E. minima*, *E. grusonii*, *E. pentacanthus*, *Gy. anisitsii*, *G. dansii*, *G. hostii*, *M. berlanensis,* and *N. leninghausii*. In addition, the three tested isolates were pathogenic on artificially inoculated plants of *As. asterias*, *Fe. glaucescens*, *Fe. macrodiscus*, *M. gracilis*, *M. spinosissima*, and *O. ficus-indica*. To the best of our knowledge, this is the first time that *N. falciformis* is reported as a pathogen of *Cactaceae* and *Crassulaceae* species worldwide.

The three fusarioid species recovered from *Cactaceae* and other succulents in Shiraz County induced diverse types of symptoms. It can be speculated that some of these symptoms, such as wilt, yellowing, and necrotic spots on the stem, might have been induced by diffusible secondary toxic metabolites of these pathogens. *Fusarium* and *Fusarium*-like species are known to produce an array of toxins, including food contaminants, usually referred to as mycotoxins, and phytotoxins, i.e., toxins that can act as pathogenicity or virulence factors in plant diseases [64,65,66,67,68]. The ability to produce toxins was also used as an accessory taxonomic criterion to separate different genotypes within *Fusarium* species complexes [69,70,71]. Moreover, toxins were demonstrated to be responsible for host-specificity of diverse ff. spp. and races of *Fusarium* [72]. The production of host-specific phytotoxins is also a distinctive trait of ff. spp. and pathotypes of other toxin-producing fungi, such as *Alternaria alternata* [73]. Interestingly, *F. proliferatum*, one of the three fusarioid species recovered from succulents in Shiraz County, was shown to produce fusaproliferin, whose deacetylated derivative, named terpestatin or siccanol, was able to induce necrosis when injected on the stem of *Opuntia ficus-indica* [74,75]. Most probably, the characterization of phytotoxins produced by *F. oxysporum* f. sp. *opuntiarum*, *F. proliferatum*, and *N. falciformis* and the study of their role in the plant-pathogen interaction might provide new insights into the pathogenesis mechanisms of Fusarium rot of *Cactaceae* and other succulent plants.

Several factors favor the spread of Fusarium rot and make the management of this disease problematic in commercial greenhouses, including the involvement of multiple *Fusarium* and *Fusarium*-like species in the disease etiology, the ability of these fungi to switch to a saprophytic lifestyle and survive in soil on plant debris, their polyphagy facilitating cross-infections and, last but not least, vegetative propagation, which is the most commonly used method to propagate succulents in commercial nurseries.

## 5. Conclusions

Overall, results of this study confirm *Fusarium* and *Fusarium*-like species are a serious constraint for the commercial production of ornamental succulents under greenhouse conditions. Although *F. oxysporum* f. sp. *opuntiarum* was the prevalent species associated with Fusarim rot of succulents in surveyed commercial greenhouses of Shiraz County, the other two fungal species also recovered from infected plants, *F. proliferatum* and *N. falciformis*, were shown to be very aggressive on a wide range of succulents. Consistently with previous studies [11], results of pathogenicity tests indicate there are differences in susceptibility to Fusarium rot among species of succulents even within the same genus, as in the case of *Mammillaria*. Consequently, it can be assumed that planting species less susceptible to Fusarium rot may be a way to prevent and reduce the damage caused by this disease in commercial cultivations. Moreover, a better insight into the ecology and epidemiology of diverse fusarioid species associated with Fusarium rot of succulents as well as the characterization of putative pathogenicity-related genes of these fungi [76,77] may be useful to implement effective disease management strategies, such as the application of safe and effective natural antifungal preparation to control the disease [78].

## Figures and Tables

**Table 1:** Fusarioid species isolates recovered from ornamental cacti and other succulent plants collected in commercial greenhouses of Shiraz County, Iran.

Species	Isolates	Collection Date	Location	Longitude	Latitude	Matrix ^a^	GenBank Accession No. ^b^
*Neocosmospora falciformis*							
	FNol01	October 2018	Bajgah	29°43′23.3″ N	52°35′30.0″ E	NoLe crown	OM801788
	FGyh01	February 2018	Ghast-e Dasht	29°39′33.0″ N	52°28′50.9″ E	GyHo stem	OM801786
	FMab01	September 2018	Ghast-e Dasht	29°39′33.0″ N	52°28′50.9″ E	MaBe crown	N/A
	FNol05	April 2018	Bajgah	29°43′23.3″ N	52°35′30.0″ E	NoLe crown	N/A
	FEcp01	September 2018	Ghast-e Dasht	29°39′27.6″ N	52°28′54.3″ E	EaPe crown	N/A
	FEcg01	September 2018	Ghast-e Dasht	29°39′31.7″ N	52°28′51.1″ E	EaGr crown	N/A
	Fgyb03	October 2018	Bajgah	29°43′23.3″ N	52°35′30.0″ E	GyDa stem	N/A
	FAeg01	February 2018	Ghast-e Dasht	29°39′27.6″ N	52°28′54.3″ E	AeGo crown	OM801787
	FGya01	February 2018	Ghast-e Dasht	29°39′27.6″ N	52°28′54.3″ E	GyAi crown	N/A
	FGya02	February 2018	Ghast-e Dasht	29°38′40.0″ N	52°28′05.2″ E	CeEu root	N/A
	FCee02	February 2018	Ghast-e Dasht	29°39′30.4″ N	52°28′52.8″ E	EaGr crown	N/A
	FEcg11	October 2018	Sadra	29°48′52.4″ N	52°29′26.0″ E	EaGr stem	N/A
	FEcg02	October 2018	Sadra	29°48′52.4″ N	52°29′26.0″ E	EaGr stem	N/A
	FEcm01	September 2018	Ghast-e Dasht	29°39′27.6″ N	52°28′54.3″ E	EcMi root	N/A
	FAeg11	September 2018	Ghast-e Dasht	29°39′31.7″ N	52°28′51.1″ E	AeGo root	N/A
	FEcg21	September 2018	Ghast-e Dasht	29°39′33.0″ N	52°28′50.9″ E	EcGi root	N/A
	FMab11	September 2018	Ghast-e Dasht	29°39′33.0″ N	52°28′50.9″ E	MaBe root	N/A
*Fusarium oxysporum* f. sp. *opuntiarum*							
	OGyf01	February 2018	Ghast-e Dasht	29°39′27.6″ N	52°28′54.3″ E	GyFe stem	OM801795
	OAsm01	February 2018	Ghast-e Dasht	29°38′40.0″ N	52°28′04.7″ E	AsMy stem	N/A
	OFel01	September 2018	Ghast-e Dasht	29°39′33.0″ N	52°28′50.9″ E	FeAl stem	N/A
	OEeh31	September 2018	Ghast-e Dasht	29°39′33.0″ N	52°28′50.9″ E	EaHo crown	N/A
	OAsm11	September 2018	Ghast-e Dasht	29°39′33.0″ N	52°28′50.9″ E	AsMy stem	N/A
	OMae04	October 2018	Bajgah	29°43′23.3″ N	52°35′30.0″ E	MaEl crown	N/A
	ONos03	October 2018	Bajgah	29°43′23.3″ N	52°35′30.0″ E	NoRu crown	OM801797
	ONos04	July 2018	Bajgah	29°43′23.3″ N	52°35′30.0″ E	NoRu crown	OM801798
	OEep02	October 2018	Bajgah	29°43′23.3″ N	52°35′30.0″ E	EePh stem	OM801793
	OEcg36	February 2018	Ghast-e Dasht	29°39′27.6″ N	52°28′54.3″ E	EaGr crown	N/A
	OMas01	September 2018	Ghast-e Dasht	29°38′40.0″ N	52°28′05.2″ E	MaSp root	N/A
	OAsm21	April 2018	Ghast-e Dasht	29°39′31.7″ N	52°28′51.1″ E	AsMy root	OM801790
	OEcg01	July 2018	Sadra	29°48′52.4″ N	52°29′26.0″ E	EaGr crown	N/A
	OEcv01	July 2018	Sadra	29°48′52.4″ N	52°29′26.0″ E	EeNi crown	N/A
	OEcp01	September 2018	Ghast-e Dasht	29°39′41.6″ N	52°28′43.6″ E	EePh root	N/A
	OEep01	October 2018	Bajgah	29°43′23.3″ N	52°35′30.0″ E	EePh stem	N/A
	OFel11	February 2018	Ghast-e Dasht	29°39′33.0″ N	52°28′50.9″ E	FeAl crown	OM801794
	OEep11	July 2018	Ghast-e Dasht	29°39′33.0″ N	52°28′50.9″ E	EaPe soil	N/A
	OEag13	July 2018	Bajgah	29°43′23.3″ N	52°35′30.0″ E	EaGr crown	N/A
	OMap01	February 2018	Ghast-e Dasht	29°39′30.4″ N	52°28′52.8″ E	MaGr crown	OM801796
	OGym01	February 2018	Ghast-e Dasht	29°39′33.0″ N	52°28′50.9″ E	MaGr crown	N/A
	OEcp11	February 2018	Ghast-e Dasht	29°38′40.0″ N	52°28′05.2″ E	EaPe root	N/A
	OEcp21	September 2018	Ghast-e Dasht	29°39′33.0″ N	52°28′50.9″ E	EaPe crown	N/A
	OAea01	October 2018	Bajgah	29°43′23.3″ N	52°35′30.0″ E	AeAr crown	N/A
	OAsm31	June 2018	Ghast-e Dasht	29°38′40.0″ N	52°28′05.2″ E	AsMy crown	OM801791
	ONol02	September 2018	Ghast-e Dasht	29°39′27.6″ N	52°28′54.3″ E	NoLe crown	N/A
	OMam01	September 2018	Ghast-e Dasht	29°38′40.0″ N	52°28′05.2″ E	MaMa stem	N/A
	OMap03	October 2018	Bajgah	29°43′23.3″ N	52°35′30.0″ E	MaPe root	N/A
	OMap04	October 2018	Bajgah	29°43′23.3″ N	52°35′30.0″ E	MaPe root	N/A
	OGyd01	July 2018	Sadra	29°48′52.4″ N	52°29′26.0″ E	GyDa stem	N/A
	OMaj01	July 2018	Sadra	29°48′52.4″ N	52°29′26.0″ E	MaJa stem	N/A
	OEcg42	February 2018	Ghast-e Dasht	29°38′40.0″ N	52°28′05.2″ E	EaGr stem	OM801792
	ONom01	February 2018	Ghast-e Dasht	29°39′30.4″ N	52°28′52.8″ E	NoMa root	N/A
	ONom02	February 2018	Ghast-e Dasht	29°39′27.6″ N	52°28′54.3″ E	NoMa root	N/A
	ONom05	February 2018	Ghast-e Dasht	29°38′40.0″ N	52°28′04.7″ E	NoMa root	N/A
	OMap05	February 2018	Ghast-e Dasht	29°39′27.6″ N	52°28′54.3″ E	MaPr root	N/A
	OMag02	February 2018	Ghast-e Dasht	29°39′30.4″ N	52°28′52.8″ E	MaGr root	N/A
	OGyd11	February 2018	Ghast-e Dasht	29°39′33.0″ N	52°28′50.9″ E	GyDa root	N/A
*Fusarium proliferatum*							
	PEcg29	February 2018	Ghast-e Dasht	29°39′27.6″ N	52°28′54.3″ E	EaGr crown	OM801789
	PEcg02	February 2018	Ghast-e Dasht	29°39′27.6″ N	52°28′54.3″ E	EaGr crown	N/A
	PFeg01	February 2018	Ghast-e Dasht	29°39′31.7″ N	52°28′51.1″ E	FeGa root	N/A
	PAsm09	February 2018	Ghast-e Dasht	29°39′30.4″ N	52°28′52.8″ E	AsMy stem	N/A
	PMap01	February 2018	Ghast-e Dasht	29°39′33.0″ N	52°28′50.9″ E	MaPr stem	N/A
	PMav02	February 2018	Ghast-e Dasht	9°39′27.6″ N	52°28′54.3″ E	MaVe crown	N/A
	PNor01	February 2018	Ghast-e Dasht	29°39′41.4″ N	52°28′43.7″ E	NoRu root	N/A

^a^ AeAr, *Aeonium arboreum* Webb and Berthel (*Crassulaceae*); AeGo, *Aeonium gomerense* Webb and Berthel (*Crassulaceae*); AsMy, *Astrophytum myriostigma* (Zucc.) Lem. (*Cactaceae*); CeEu, *Cephalocereus euphorbioides* (Haw.) Britton and Rose, syn. *Neobuxbaumia euphorbioides* (Haw.) Buxb. (*Cactaceae*); EaGr, *Echinocactus grusonii* Hildm. (*Cactaceae*); EaHo, *Echinocactus horizonthalonius* Lem (*Cactaceae*); EaPe, * Echinocactus pentacanthus* Lem (*Cactaceae*); EcGi, *Echeveria gibbiflora* DC (*Crassulaceae*); EcMi, *Echeveria minima* Meyran (*Crassulaceae*); EeNi, *Echinocereus nivosus* Foster and Glass (*Cactaceae*); EePh, *Echinocereus pulchellus* (Mart.) K. Schum. (*Cactaceae*); FeAl, *Ferocactus alamosanus* Britton and Rose (*Cactaceae*); FeGa, *Ferocactus gatesii* Lindsay (*Cactaceae*); GyAi, *Gymnocalycium anisitsii* Britton and Rose (*Cactaceae*); GyDa, *Gymnocalycium damsii* Schumann (*Cactaceae*); GyFe, *Gymnocalycium ferox* Backeb (*Cactaceae*); GyHo, *Gymnocalycium horstii* Buining (*Cactaceae*); MaBe, *Mammillaria bernalensis* Reppen (*Cactaceae*); MaEl, *Mammillaria elongata* de Candolle (*Cactaceae*); MaGr, *Mammillaria gracilis* (*Cactaceae*); MaJa, *Mammillaria jaliscana* Britton and Rose (*Cactaceae*); MaMa, *Mammillaria matudae* Bravo (*Cactaceae*); MaPe, *Mammillaria petersonii* Hildm (*Cactaceae*); MaPr, *Mammillaria prolifera* Haworth (*Cactaceae*); MaSp, *Mammillaria spinosissima* Lemaire (*Cactaceae*); MaVe, *Mammillaria vetula* Martius (*Cactaceae*); NoLe, *Notocactus leninghausii* Brandt, syn. *Parodia leninghausii* (Schumann) Brandt (*Cactaceae*); NoMa, *Notocactus mammulosus* (Lem.) Backeb., syn. *Parodia mammulosa* (Lemaire) N.P. Taylor (*Cactaceae*); NoRu, *Notocactus rutilans* Abraham, syn. *Parodia rutilans* (Däniker and Krainz) N.P. Taylor (*Cactaceae*). ^b^ Translation elongation factor 1-α (*tef1*) gene.

**Table 2:** Species of succulent plants that proved to be susceptible to three fusarioid fungal species in pathogenicity tests. Two diverse artificial inoculation methods were used: wound inoculation with a conidial suspension (10^6^ conidia mL^−1^) and inoculation through the soil with infested wheat grains as inoculum.

Fungal Species		
*Neocosmospora falciformis*	*Fusarium oxysporum* f. sp. *opuntiarum*	*Fusarium proliferatum*
**Test Plants**		
*Aeonium arboreum* (L.) Webb and Berthel	*Aeonium arboreum* (L.) Webb and Berthel	*Astrophytum myriostigma* (Zucc.) Lem.
*Astrophytum asterias* (Zucc.) Lem.	*Astrophytum asterias* (Zucc.) Lem.	*Cephalocereus euphorbioides* Britton and Rose
*Echinocactus grusonii* Hildm.	*Astrophytum myriostigma* (Zucc.) Lem.	*Cereus jamacaru* DC.
*Ferocactus glaucescens* (DC.) Britton and Rose	*Braunsia apiculata* (Kensit) L. Bolus	*Echinocactus grusonii* Hildm.
*Ferocactus macrodiscus* Britton and Rose	*Carnegiea polylopha* Hunt (syn. *Neobuxbaumia polylopha* (DC.) Bauckeberg)	*Mammillaria gracilis* Pfeiff. *Mammillaria jaliscana* Britton and Rose
*Mammillaria bernalensis* Repp.	*Cereus jamacaru* DC.	*Mammillaria pottsii* Scheer ex Salm–Dyck
*Mammillaria gracilis* Pfeiff.	*Echinocactus grusonii* Hildm	*Mammillaria prolifera* (Mill.) Haw.
*Mammillaria prolifera* (Mill.) Haw.	*Echinocereus nivosus* Glass and Foster	
*Mammillaria spinosissima* Lem.	*Ferocactus emoryi* Orcutt	
*Opuntia ficus-indica* (L.) Mill.	*Ferocactus foetens* Britton and Rose	
	*Ferocactus glaucescens* Britton and Rose	
	*Ferocactus macrodiscus* Britton and Rose	
	*Gymnocalycium mihanovichii* Fric ex Gürke) Britton & Rose	
	*Hamatocactus setispinus* (Engelm.) Britton and Rose	
	*Mammillaria gracilis* Pfeiff.	
	*Mammillaria jaliscana* Britton and Rose	
	*Mammillaria matudae* Bravo	
	*Mammillaria pottsii* Scheer ex Salm–Dyck	
	*Mammillaria spinosissima* (Kuntze) Lem.	
	*Opuntia fragilis* (Nutt.) Haw.	
	*Sedum angelina* L.	

## Data Availability

Not applicable.

## References

[1] Eggli U. (2004). Illustrated Handbook of Succulent Plants: Dicotyledons.

[2] Albers F., Meve U., Albers F., Meve U. (2002). Asclepiadaceae. Illustrated Handbook of Succulent Plants: Aizoaceae A–E..

[3] Hartmann K.E.K., Hartmann K.E.K. (2002). Aizoaceae. Illustrated Handbook of Succulent Plants: Aizoaceae A–E..

[4] Eggli U., Eggli U. (2003). Crassulaceae. Illustrated Handbook of Succulent Plants: Crassulaceae.

[5] Charles G. (2014). Cacti and Succulents: An Illustrated Guide to the Plants and Their Cultivation.

[6] Christenhusz M.J., Byng J.W. (2016). The number of known plant species in the world and its annual increase. Phytotaxa.

[7] Kaplan H., Wilson J.R.U., Klein H., Henderson L., Zimmermann H.G., Manyama P., Ivey P., Richardson D.M., Novoa A. (2017). A proposed national strategic framework for the management of *Cactaceae* in South Africa. Bothalia.

[8] Ochoa M.J., Barbera G., Inglese P., Mondragon C., Nefzaoui A., Saenz C. (2017). History and Economic and Agro-Ecological Importance. Crop Ecology, Cultivation and Uses of Cactus Pear.

[9] Wick R.L., McGovern R., Elmer W. (2017). Diseases of Holiday Cacti: *Schlumbergera* and *Hatiora*. Handbook of Florists’ Crops Diseases, Handbook of Plant Disease Management Series.

[10] Aloi F., Giambra S., Schena L., Surico G., Pane A., Gusella G., Stracquadanio C., Burruano S., Cacciola S.O. (2020). New insights into scabby canker of *Opuntia ficus-indica*, caused by *Neofusicoccum batangarum*. Phytopathol. Mediterr..

[11] Bertetti D., Ortu G., Gullino M.L., Garibaldi A. (2017). Identification of *Fusarium oxysporum* f. sp. *opuntiarum* on new hosts of the Cactaceae and Euphorbiaceae families. J. Plant Pathol..

[12] Lombard L., van der Merwe N.A., Groenewald J.Z., Crous P. (2015). Generic concepts in *Nectriaceae*. Stud. Mycol..

[13] Sandoval-Denis M., Lombard L., Crous P. (2019). Back to the roots: A reappraisal of *Neocosmospora*. Persoonia.

[14] O’Donnell K. (2000). Molecular phylogeny of the *Nectria haematococca*–*Fusarium solani* species complex. Mycologia.

[15] Geiser D.M., Aoki T., Bacon C.W., Baker S.E., Bhattacharyya M.K., Brandt M.E., Brown D.W., Burgess L.W., Chulze S., Coleman J.J. (2013). One fungus, one name: Defining the genus *Fusarium* in a scientifically robust way that preserves longstanding use. Phytopathology.

[16] O’Donnell K., Al-Hatmi A.M.S., Aoki T., Brankovics B., Cano-Lira J.F., Coleman J.J., de Hoog G.S., Di Pietro A., Frandsen R.J.N., Geiser D.M. (2020). No to *Neocosmospora: Phylogenomic* and practical reasons for continued inclusion of the *Fusarium solani* species complex in the genus *Fusarium*. Mycosphere.

[17] Edel-Hermann V., Lecomte C. (2019). Current Status of *Fusarium oxysporum Formae Speciales* and Races. Phytopathology.

[18] Hawa M., Salleh B., Latiffah Z. (2013). Characterization and pathogenicity of *Fusarium proliferatum* causing stem rot of *Hylocereus polyrhizus* in Malaysia. Ann. Appl. Biol..

[19] Ortu G., Bertetti D., Gullino M.L., Garibaldi A. (2013). A new *forma specialis* of *Fusarium oxysporum* on *Crassula ovata*. J. Plant Pathol..

[20] Ortu G., Bertetti D., Gullino M.L., Garibaldi A. (2015). *Fusarium oxysporum* f. sp. *echeveriae*, a novel forma specialis causing crown and stem rot of Echeveria agavoides. Phytopathol. Mediterr..

[21] Masratul Hawa M., Nurul Faziha I., Nik Mohamad Izham M.N., Latiffah Z. (2017). *Fusarium fujikuroi* associated with stem rot of red-fleshed dragon fruit (*Hylocereus polyrhizus*) in Malaysia *Ann*. Appl. Biol..

[22] Wright E.R., Rivera M.C., Ghirlanda A., Lori G.A. (2007). Basal rot of *Hylocereus undatus* caused by *Fusarium oxysporum* in Buenos Aires, Argentina. Plant Dis..

[23] Vakalounakis D.J., Kavroulakis N., Lamprou K. (2015). First report of *Fusarium oxysporum* causing root and crown rot on barbados aloe in Greece. Plant Dis..

[24] Ramírez-Ramírez M., Mancilla-Margalli N.A., Meza-Álvarez L., Turincio-Tadeo R., Guzmán-de Pena D., Avila-Miranda M.E. (2017). Epidemiology of Fusarium agave wilt in *Agave tequilana* Weber var. azul. Plant Protect. Sci..

[25] Ogórek R., Piecuch A., Kędzior M. (2021). *Fusarium oxysporum* as a pathogen of pot plants: A case study of the easter lily cactus (*Echinopsis oxygona*) in Poland. Pol. J. Environ. Stud..

[26] Bertetti D., Pensa P., Matic S., Gullino M.L., Garibaldi A. (2020). Stem rot caused by *Fusarium oxysporum* f. sp. *opuntiarum* on *Mammillaria painteri* in Italy. Phytopathol. Mediter..

[27] Safaiefarahani B., Mostowfizadeh-Ghalamfarsa R. (2014). Identification and morphological characterization of *Fusarium oxysporum* f. sp. *opuntiarum*, the causal agent of basal stem rot of cactus in Fars province. Iran J. Plant Pathol..

[28] Jeffers S.N., Martin S.B. (1986). Comparision of two media selective for *Phytophthora* and *Pythium* species. Plant Dis..

[29] Choi Y.W., Hyde K.D., Ho W.H. (1999). Single spore isolation of fungi. Fungal Divers..

[30] Aoki T., O’Donnell K., Homma Y., Lattanzi A.R. (2003). Sudden-death syndrome of soybean is caused by two morphologically and phylogenetically distinct species within the *Fusarium solani* species complex—*F. virguliforme* in North America and *F. tucumaniae* in South America. Mycologia.

[31] Aoki T., O’Donnell K., Scandiani M.M. (2005). Sudden death syndrome of soybean in South America is caused by four species of *Fusarium*: *Fusarium brasiliense* sp. nov., *F. cuneirostrum* sp. nov., *F. tucumaniae* and *F. virguliforme*. Mycologia.

[32] Leslie J.F., Summerell B.A. (2006). The Fusarium Laboratory Manual.

[33] Thompson R.S., Aveling T.A.S., Blanco Prieto R. (2013). A new semi-selective medium for *Fusarium graminearum*, *F. proliferatum*, *F. subglutinans* and *F. verticillioides* in maize seed. S. Afr. J. Bot..

[34] Fisher N.L., Burgess L.W., Toussoun T.A., Nelson P.E. (1982). Carnation leaves as a substrate and for preserving cultures of *Fusarium* species. Phytopathology.

[35] Yilmaz N., Sandoval-Denis M., Lombard L., Visagie C.M., Wingfield B.D., Crous P.W. (2021). Redefining species limits in the *Fusarium fujikuroi* species complex. Persoonia.

[36] Nelson P.E., Toussoun T.A., Cook R.J. (1982). Fusarium: Diseases, Biology and Taxonomy.

[37] Schena L., Abdelfattah A., Mosca S., Li Destri G., Agosteo G.E., Cacciola S.O. (2017). Quantitative detection of *Colletotrichum godetiae* and *C. acutatum sensu stricto* in the phyllosphere and carposphere of olive during four phenological phases. Eur. J. Plant Pathol..

[38] O’Donnell K., Sutton D.A., Rinaldi M.G., Sarver B.A., Balajee S.A., Schroers H.J., Summerbel R.C., Varg R., Crous P.W., Zhang N. (2010). Internet-accessible DNA sequence database for identifying *Fusaria* from human and animal infections. J. Clin. Microbiol..

[39] Hall T.A. (1999). BioEdit: A user-friendly biological sequence alignment editor and analysis program for Windows 95/98/NT. Nucleic Acids Symposium Series.

[40] Thompson J.D., Gibson T.J., Plewniak F., Jeanmougin F., Higgins D.G. (1997). The ClustalX windows interface: Flexible strategies for multiple sequence alignment aided by quality analysis tools. Nucleic Acids Res..

[41] Ronquist F., Huelsenbeck J.P. (2003). MrBayes 3: Bayesian phylogenetic inference under mixed models. Bioinformatics.

[42] Salmaninezhad F., Mostowfizadeh-Ghalamfarsa R. (2019). Three new *Pythium* species from rice paddy fields. Mycologia.

[43] Nylander J.A., Ronquist F., Huelsenbeck J.P., Nieves-Aldrey J. (2004). Bayesian phylogenetic analysis of combined data. Systematic Biol..

[44] Mishra B., Ploch S., Weiland C., Thines M. TrEase—A Webserver to Infer Phylogenetic Trees with Ease. http://www.thines-lab.senckenberg.de/trease.

[45] Stöver B.C., Müller K.F. (2010). TreeGraph 2: Combining and visualizing evidence from different phylogenetic analyses. BMC Bioinform..

[46] TreeBASE. http://www.treebase.org.

[47] Sabahi F. (2013). Invenstivigation of Fungal and Fungal-like Soil Borne Pathogens Causal Agents of Oranamentals Mortality in Shiraz. Master’s Thesis.

[48] Westerlund F.V., Campbell R.N., Kimble K.A. (1974). Fungal root rots and wilt of chickpea in California. Phytopathology.

[49] Gerlach W. (1972). Fusarium rot and other fungal diseases of horticulturally important cacti in Germany. Phytopathol. Z..

[50] Van Dam P., Fokkens L., Schmidt S.M., Linmans J.H., Kistler H.C., Ma L.J., Rep M. (2016). Effector profiles distinguish formae speciales of *Fusarium oxysporum*. Environ. Microbiol..

[51] Lievens B., Hanssen I.M., Rep M., Gullino M.L., Katan J., Garibaldi A. (2017). Recent developments in the detection and identification of formae speciales and races of *Fusarium oxysporum*: From pathogenicity testing to molecular diagnostics. Fusarium Wiltings of Vegetable and Ornamental Crops.

[52] Duan Y., Qu W., Chang S., Li C., Xu F., Ju M., Zhao R., Wang H., Zhang H., Miao H. (2020). Identification of pathogenicity groups and pathogenic molecular characterization of *Fusarium oxysporum* f. sp. *Sesame* in China. Phytopathology.

[53] O’Donnell K., Kistler H.C., Cigelnik E., Ploetz R.C. (1998). Multiple evolutionary origin of the fungus causing panama disease of banana: Concordant evidence from nuclear and mitochondrial gene genealogies. Proc. Nat. Acad. Sci. USA.

[54] Baayen R.P., O’Donnell K., Bonants P.J.M., Cigelnik E., Kroon L.P.N.M., Roebroeck E.J.A., Waalwijk C. (2000). Gene genealogies and AFLP analyses in the *Fusarium oxysporum* complex identify monophyletic and non-monophyletic formae speciales causing wilt and rot disease. Phytopathology.

[55] Skovgaard K., Nirenberg H.I., O’Donnell K., Rosendahl S. (2001). Evolution of *Fusarium oxysporum* f. sp. *vasinfectum* races inferred from multigene genealogies. Phytopathology.

[56] Chehri K. (2017). Molecular identification of entomopathogenic *Fusarium* species associated with *Tribolium* species in stored grains. J. Invertebr. Pathol..

[57] Gwinn K.D., Hansen Z., Kelly H., Ownley B.H. (2022). Diseases of *Cannabis sativa* caused by diverse *Fusarium* species. Front. Agron..

[58] Narmani A., Arzanlou M., Babai Ahari A., Hatef H., Ghasemi S. (2016). First report of fungal soft rot disease caused by *Fusarium proliferatum* on peanut cactus (*Echinopsis chamaecereus*) in the world. Proceedings of the 22nd Iranian Plant Protection Congress, College of Agriculture and Natural Resources.

[59] O’Donnell K., Sutton D.A., Fothergill A., McCarthy D., Rinaldi M.G., Brandt M.E., Zhang N., Geiser D.M. (2008). Molecular phylogenetic diversity, multilocus haplotype nomenclature, and in vitro antifungal resistance within the *Fusarium solani* species complex. J. Clin. Microbiol..

[60] Geiser D.M., Al-Hatmi A.M.S., Aoki T., Arie T., Balmas V., Barnes I., Bergstrom G.C., Bhattacharyya M.K., Blomquist C.L., Bowden R.L. (2021). Phylogenomic analysis of a 55.1 kb 19-gene dataset resolves a monophyletic *Fusarium* that includes the *Fusarium solani* species complex. Phytopathology.

[61] Costa S.S., Matos K.S., Tessmann D.J., Seixas C.D., Pfenning L.H. (2016). *Fusarium paranaense* sp. nov., a member of the *Fusarium solani* species complex causes root rot on soybean in Brazil. Fungal Biol..

[62] Sousa E.S., Melo M.P., Mota J.M., Sousa E.M.J., Beserra J.E.A., Matos K.S. (2017). First report of *Fusarium falciforme* (FSSC 3 + 4) causing root rot in lima bean (*Phaseolus lunatus* L.) in Brazil. Plant Dis..

[63] López-Bautista V., Mora-Aguilera G., Gutiérrez-Espinosa M.A., Mendoza-Ramos C., Martínez-Bustamante V.I., Coria-Contreras J.J., Acevedo-Sánchez G., Santana-Peñaloza B. (2020). Morphological and molecular characterization of *Fusarium* spp. associated to the regional occurrence of wilt and dry bud rot in *Agave tequilana*. Mex. J. Phytopathol..

[64] Nesic K., Ivanovic S., Nesic V. (2014). Fusarial toxins: Secondary metabolites of *Fusarium* fungi. Rev. Environ. Contam. Toxicol..

[65] Chang H.-X., Domier L.L., Radwan O., Yendrek C.R., Hudson M.E., Hartman G.L. (2016). Identification of multiple phytotoxins produced by *Fusarium virguliforme* including a phytotoxic effector (FvNIS1) associated with sudden death syndrome foliar symptoms. MPMI.

[66] Perincherry L., Lalak-Kańczugowska J., Stępień Ł. (2019). *Fusarium*-produced mycotoxins in plant-pathogen interactions. Toxins.

[67] Bentivenga G., Spina A., Ammar K., Allegra M., Cacciola S.O. (2021). Screening of durum wheat (*Triticum turgidum* L. subsp. *durum* (Desf.) Husn.) Italian cultivars for susceptibility to fusarium head blight incited by *Fusarium graminearum*. Plants.

[68] Stracquadanio C., Luz C., La Spada F., Meca G., Cacciola S.O. (2021). Inhibition of mycotoxigenic fungi in different vegetable matrices by extracts of *Trichoderma* Species. J. Fungi.

[69] Somma S., Petruzzella A.L., Logrieco A.F., Meca G., Cacciola S.O., Moretti A. (2014). Phylogenetic analyses of *Fusarium graminearum* strains from cereals in Italy, and characterisation of their molecular and chemical chemotypes. Crop Pasture Sci..

[70] Nirmaladevi D., Venkataramana M., Srivastava R., Uppalapati S.R., Gupta V.K., Yli-Mattila T., Tsui K.M.C., Srinivas C., Niranjana S.R., Chandra N.S. (2016). Molecular phylogeny, pathogenicity and toxigenicity of *Fusarium oxysporum* f. sp. *lycopersici*. Sci. Rep..

[71] Pasquali M., Beyer M., Logrieco A., Audenaert K., Balmas V., Basler R., Boutigny A.-L., Chrpová J., Czembor E., Gagkaeva T. (2016). A European database of *Fusarium graminearum* and *F. culmorum* trichothecene genotypes. Front. Microbiol..

[72] Sutherland M.L., Pegg G.F. (1995). Purification of a toxin from *Fusarium oxysporum* f. sp. *lycopersici* race 1. Physiol. Mol. Plant Pathol..

[73] Aloi F., Riolo M., Sanzani S.M., Mincuzzi A., Ippolito A., Siciliano I., Pane A., Gullino M.L., Cacciola S.O. (2021). Characterization of *Alternaria* Species Associated with Heart Rot of Pomegranate Fruit. J. Fungi.

[74] Masi M., Aloi F., Nocera P., Cacciola S.O., Surico G., Evidente A. (2020). Phytotoxic metabolites isolated from *Neofusicoccum batangarum*, the causal agent of the scabby canker of cactus pear (*Opuntia ficus-indica* L.). Toxins.

[75] Ćeranić A., Svoboda T., Berthiller F., Sulyok M., Samson J.M., Güldener U., Schuhmacher R., Adam G. (2021). Identification and functional characterization of the gene cluster responsible for fusaproliferin biosynthesis in *Fusarium proliferatum*. Toxins.

[76] Lievens B., Houterman P.M., Rep M. (2009). Effector gene screening allows unambiguous identification of *Fusarium oxysporum* f. sp. *lycopersici* races and discrimination from other formae speciales. FEMS Microbiol. Lett..

[77] Taylor A., Vágány V., Jackson A.C., Harrison R.J., Rainoni A., Clarkson J.P. (2015). Identification of pathogenicity-related genes in *Fusarium oxysporum* f. sp. *cepae*. Mol. Plant Pathol..

[78] Pangallo S., Li Destri Nicosia M.G., Agosteo G.E., Abdelfattah A., Romeo F.V., Cacciola S.O., Rapisarda P., Schena L. (2017). Evaluation of a pomegranate peel extract (PGE) as alternative mean to control olive anthracnose. Phytopathology.

